# Classification model of amino acid sequences prone to aggregation of therapeutic proteins

**DOI:** 10.1186/s40203-016-0019-4

**Published:** 2016-07-07

**Authors:** Monika Marczak, Krystyna Okoniewska, Tomasz Grabowski

**Affiliations:** Dofarm, ul. Aksamitna 15, 05-870 Błonie, Poland; P.F.O. Vetos-Farma sp. z o. o., ul. Dzierżoniowska 21, 58-260 Bielawa, Poland; Polpharma Biologics, ul.Trzy lipy 3, 80-172 Gdańsk, Poland

**Keywords:** *In silico*, ADA, Aggregates, Proteins, Immunogenicity

## Abstract

**Background:**

Total body clearance of biological drugs is for the most part dependent on the receptor mechanisms (receptor mediated clearance) and the concentration of antibodies aimed at administered drug – anti-drug-antibodies (ADA). One of the significant factors that induces the increase of ADA level after drug administration could be the aggregates present in the finished product or formed in the organism. Numerous attempts have been made to identify the sequence fragments that could be responsible for forming the aggregates – aggregate prone regions (APR).

**Purpose:**

The aim of this study was to find physiochemical parameters specific to APR that would differentiate APR from other sequences present in therapeutic proteins.

**Methods:**

Two groups of amino acid sequences were used in the study. The first one was represented by the sequences separated from the therapeutic proteins (*n* = 84) able to form APR. A control set (CS) consisted of peptides that were chosen based on 22 tregitope sequences.

**Results:**

Classification model and four classes (A, B, C, D) of sequences were finally presented. For model validation Cooper statistics was presented.

**Conclusions:**

The study proposes a classification model of APR. This consists in a distinction of APR from sequences that do not form aggregates based on the differences in the value of physicochemical parameters. Significant share of electrostatic parameters in relation to classification model was indicated.

## Background

Therapeutic proteins are one of the fastest developing types of drug. A pharmacokinetic profile as well as the effect of biological drugs are the result of many complex interactions with the immune system (Mould and Green 2010; Dostalek et al. 2012). One of the key pharmacokinetic parameters of biological drugs is clearance (EMA 2012). Total body clearance of biological drugs is for the most part dependent on the receptor mechanisms (receptor mediated clearance) and the concentration of antibodies aimed at the administered drug – anti-drug-antibodies (ADA) (Datta-Mannan et al. 2007; Wang and Chow 2010). ADA are produced in the organism as a response to most of the biological drugs including humanized molecules and completely human monoclonal antibodies. The issue of immune response to biological drugs is treated by the health authorities (FDA 2014). Apart from binding (binding antibodies), ADA can also neutralize drugs present in the organism (neutralizing antibodies) (Hsu et al. 2014). One of the significant factors that induces the increase of ADA level after drug administration could be the aggregates present in the finished product or formed in the organism (Chennamsetty et al. 2009; Wang et al. 2009). Protein aggregations, depending on their structure, can exhibit different immunogenicity. Even a slight quantity of formed aggregations after an administration of biological drug may induce a significant increase of ADA level (Rosenberg 2006). Insufficient knowledge about the possibility of aggregation process induction at the stage of drug design may endanger safety and efficacy of biological drug application in the clinical phase. However, the impact of aggregation on safety during biological drug research applications may often be difficult to predict. The reason for this is the specificity of the immune response. In extreme cases the dynamics of this response are extraordinarily high and the equivalent is not seen in small molecules.

Numerous attempts have been made to identify the sequence fragments of different proteins that could be responsible for forming the aggregates (Pawar et al. 2005; Tartaglia et al. 2005). In the case of therapeutic proteins, it is known that these fragments are short, hydrophobic sequences (aggregation prone regions – APR) that in favorable conditions initiate the aggregation process (Wang et al. 2009). A large number of APR in protein sequences could be connected with higher ability to form aggregates *in vivo*. This, in turn, can have a significant impact on the concentration of free form drug in blood and number of side effects (Rosenberg 2006). The aggregates in finished drugs are usually identified with the use of physicochemical methods such as: size exclusion chromatography, analytical ultracentrifugation, electrophoresis, light scattering etc. However, these methods have some limitations (Tatkiewicz et al. 2015). They are not always sensitive enough or they do not determine the share of aggregates of various structures in a single sample. Most of the commonly used methods allow the determination of hydrodynamic size or the molecular weight of an aggregate. These measurements ascertain or exclude the presence of aggregates only after their formation. They do not identify a danger connected with the ability of determined protein to form the aggregates. Hence, the significant tool to assess the risk relating to the ability of forming the aggregates is *in silico* analysis (Agrawal et al. 2011). Currently, different kinds of software based on phenomenological methods, statistical models, Monte Carlo simulations, scoring matrices, decision trees, Bayesian models etc. (Wang et al. 2009; Tsolis et al. 2013) are used to find APR in the sequences of therapeutic proteins.

The aim of this study was to find physiochemical parameters specific to APR that would differentiate APR from other sequences present in therapeutic proteins.

## Methods

### Sequences selection

Two groups of amino acid sequences were used in the study. The first one was represented by the sequences separated from the therapeutic proteins (*n* = 84) able to form aggregation bridges – APR (Wang et al. 2009) (Table 1; sequences 1–84). A control set (CS) consisted of peptides that were chosen based on 22 tregitope sequences (Epivax Inc. 2007).

**Table 1:** Aggregation prone regions (no. 1–84), tregitope sequences (no. 85–106) and short sequences extracted from tregitopes - control set (no. 107–149)

No.	Sequence	AM	AC	ROT	HBA	QPCaco [nm/s]	IP [eV]	NON	QPlogS	AEx
1	ALLVN	4	4	15	13.50	4.41	39.05	0	−0.012	−1.19
2	ALVLIAFA	5	5	19	15.00	7.17	48.36	0	−0.163	−3.22
3	ALYLV	4	4	16	12.75	4.76	29.11	0	−0.119	−1.10
4	CQQYN	5	5	23	21.75	1.71	38.59	0	0.174	2.69
5	DDHYC	5	7	21	19.25	2.08	38.91	0	−0.105	2.65
6	ELLFFAK	7	7	28	21.00	5.73	57.80	0	−0.082	−0.75
7	FAAFV	3	3	11	9.00	4.20	28.86	0	−0.096	−2.23
8	FALFFTIF	7	7	28	21.70	8.87	67.56	0	−0.226	−3.96
9	FAVWG	4	4	13	12.00	4.25	37.33	0	−0.098	−1.33
10	FILFAVF	6	6	23	18.00	8.46	57.82	0	−0.205	−4.01
11	FLSVFFSG	8	8	29	25.40	8.38	77.56	0	−0.122	−3.29
12	FVQWLM	6	6	25	21.00	6.24	56.03	0	−0.124	−1.63
13	GLALL	4	4	14	12.00	4.97	38.83	0	−0.088	−1.92
14	GLLYC	5	5	19	15.25	5.09	38.51	0	−0.077	−0.97
15	GSFFL	5	5	18	15.70	5.18	48.43	0	−0.080	−1.34
16	GSFFLY	6	6	23	19.45	5.60	48.43	0	−0.110	−1.04
17	GSFFLYS	7	7	27	23.15	5.96	58.29	0	−0.095	−1.04
18	IAALL	3	3	12	9.00	4.34	29.09	0	−0.106	−2.04
19	IFLFG	5	5	18	15.00	6.27	48.24	0	−0.130	−2.43
20	IFTDF	5	6	20	16.70	4.72	48.68	0	−0.147	−0.39
21	IFYFYGTTY	9	9	37	30.65	7.07	58.21	0	−0.184	−1.42
22	IGAIY	4	4	15	12.75	3.95	29.02	0	−0.083	−0.39
23	IGYIS	5	5	19	16.45	4.31	38.87	0	−0.068	−0.20
24	IGYIY	5	5	20	16.50	4.36	29.02	0	−0.113	−0.03
25	IMVTF	5	5	20	16.20	5.91	47.81	0	−0.125	−1.86
26	ISLLLIQ	7	7	29	24.20	7.63	67.96	0	−0.102	−2.68
27	IVTCVVV	7	7	24	21.20	8.93	67.63	0	−0.103	−4.25
28	IYYCV	5	5	21	16.00	4.88	28.73	0	−0.108	−0.48
29	LAILT	4	4	16	12.70	4.83	38.93	0	−0.106	−1.47
30	LFNIA	4	4	16	13.50	4.32	38.90	0	−0.032	−0.95
31	LFVEF	5	6	20	17.00	5.67	48.29	0	−0.132	−1.33
32	LGIYF	5	5	19	15.75	5.32	38.65	0	−0.122	−1.20
33	LGLLG	5	5	16	15.00	5.61	48.53	0	−0.070	−2.02
34	LGQFLLFC	8	8	31	26.00	8.90	77.02	0	−0.100	−3.72
35	LGVIW	5	5	17	15.00	5.77	47.12	0	−0.130	−2.02
36	LIGALLV	6	6	21	18.00	7.88	58.18	0	−0.142	−3.58
37	LLIYAA	4	4	17	12.75	4.75	29.09	0	−0.136	−0.99
38	LLIYAASYL	7	7	30	23.20	6.97	48.66	0	−0.187	−1.85
39	LLIYGA	5	5	19	15.75	5.39	38.78	0	−0.118	−1.28
40	LLIYSASFLY	9	9	38	29.90	8.70	68.10	0	−0.211	−3.06
41	LLMLL	5	5	21	15.50	6.91	47.90	0	−0.175	−2.78
42	LMVFFGN	7	7	26	23.00	7.47	67.24	0	−0.069	−2.67
43	LVFFA	4	4	15	12.00	5.65	38.57	0	−0.131	−2.41
44	LVYGA	4	4	14	12.75	3.96	29.09	0	−0.066	−0.53
45	NLFLLS	6	6	24	20.20	6.12	58.52	0	−0.053	−1.60
46	NVILFSVF	8	8	30	26.20	8.97	77.71	0	−0.093	−3.83
47	RGFFY	5	5	22	17.75	3.85	37.66	0	−0.091	0.50
48	SFFLY	5	5	21	16.45	4.96	38.74	0	−0.128	−0.69
49	SFFLYS	6	6	25	20.15	5.32	48.60	0	−0.113	−0.64
50	SVFIF	5	5	19	15.70	6.01	48.38	0	−0.116	−2.06
51	SVFIFP	6	6	20	19.20	7.90	57.74	4	−0.128	0.32
52	SVFLFP	6	6	20	19.20	7.90	57.79	4	−0.128	0.32
53	SVFLFPP	7	7	21	22.70	9.80	67.16	8	−0.140	2.67
54	TEYNQ	5	6	23	22.45	1.05	39.13	0	0.108	3.60
55	TLFLVY	6	6	24	19.45	6.63	48.54	0	−0.157	−2.01
56	TLLIIFK	8	7	31	22.70	7.73	67.67	0	−0.113	−2.67
57	TNYNQ	5	5	22	21.95	1.08	39.35	0	0.186	3.25
58	TTEYN	5	6	22	20.65	1.50	39.34	0	0.048	3.08
59	TVFIFP	6	6	20	19.20	8.04	57.73	4	−0.142	0.18
60	VAYWYILFIG	9	9	35	28.50	9.42	66.37	0	−0.264	−3.87
61	VEALYL	5	6	21	17.75	4.79	38.83	0	−0.119	−0.27
62	VFLGMFLY	8	8	31	25.25	9.28	67.02	0	−0.212	−4.04
63	VLIYF	5	5	20	15.75	6.14	38.65	0	−0.158	−1.94
64	VLMISL	6	6	24	19.20	7.29	57.67	0	−0.143	−2.76
65	VTLFF	5	5	19	15.70	6.14	48.41	0	−0.131	−2.20
66	VTMLV	5	5	19	16.20	6.00	47.97	0	−0.105	−2.04
67	VVCFL	5	5	18	14.50	6.88	48.06	0	−0.105	−3.03
68	VVCLL	5	5	18	14.50	6.95	48.19	0	−0.102	−3.11
69	VVITL	5	5	18	15.70	6.31	48.59	0	−0.107	−2.47
70	VVSVLTVL	8	8	28	25.40	9.59	77.87	0	−0.129	−4.55
71	VVSVLTVV	8	8	27	25.40	9.60	77.84	0	−0.111	−4.62
72	VVYYSNSYWYF	11	11	46	38.90	7.51	66.98	0	−0.147	−1.40
73	YCLQYD	6	7	27	22.50	3.50	38.73	0	−0.063	1.55
74	YCQQHNE	7	8	32	31.75	2.19	57.83	0	0.162	3.13
75	YCQQHY	6	6	28	26.00	2.53	38.17	0	0.056	2.39
76	YCQQNNN	7	7	31	30.75	1.82	58.47	0	0.329	3.28
77	YCQQS	5	5	23	20.95	2.01	38.51	0	0.112	2.39
78	YCQQYS	6	6	28	24.70	2.43	38.51	0	0.082	2.48
79	YFIAAV	4	4	16	12.75	4.69	28.93	0	−0.122	−1.03
80	YIFSNYWIQWV	11	11	46	39.20	8.59	84.76	0	−0.153	−2.52
81	YISQFIIMY	9	9	40	32.20	8.08	67.10	0	−0.163	−2.37
82	YSVVLLL	7	7	27	22.45	8.03	58.37	0	−0.158	−3.10
83	YVWQVL	6	6	24	21.25	5.61	47.09	0	−0.100	−0.98
84	YYWTWI	6	6	26	20.20	4.34	36.24	0	−0.213	0.49
85	LMIYEVSNRPSGVPD	15	17	56	55.15	12.68	134.38	8	−0.105	2.04
86	NTLYLQMNSLRAEDTAVYYCA	18	20	80	68.85	10.95	144.82	0	−0.047	−3.65
87	NSTYRVVSVLTVLH	14	14	56	51.05	11.43	125.82	0	−0.079	−4.92
88	PAVLQSSGLYSLSSVVTVPSSSLGTQ	25	25	89	88.75	21.08	233.51	8	0.009	−5.47
89	VDTSKNQFSLRLSSVTAADTA	19	20	77	67.90	10.42	175.19	0	0.049	−3.18
90	KVYACEVTHQGLSS	14	14	54	48.85	8.39	116.08	0	0.067	−1.91
91	MHWVRQAPGKGLEWV	15	15	56	52.50	10.90	130.77	4	−0.085	−0.31
92	LNNFYPREAKVQWKVDNALQSGNS	24	24	94	86.65	12.22	202.03	4	0.330	−0.58
93	VHWYQQKPGQAPVL	14	13	49	51.25	10.67	113.79	8	0.071	3.62
94	GTDFTLTISSLQPED	15	18	57	55.50	10.60	146.32	4	−0.116	0.20
95	PGLVRPSQTLSLTCT	15	15	55	53.50	13.68	144.30	8	−0.034	0.86
96	GGLVQPGGSLRLSCAASGFTF	19	19	66	64.30	16.45	183.05	4	−0.014	−5.46
97	WSWIRQPPGKGLEWI	16	16	58	54.20	12.32	139.75	8	−0.130	2.37
98	VSWYQQLPGTAPKL	14	13	49	48.15	11.01	114.36	8	0.020	3.28
99	WSWVRQPPGRGLEWI	15	16	57	55.20	12.31	139.09	8	−0.181	2.36
100	GGLVQPGRSLRLSCAASGFTF	19	19	71	66.30	15.86	182.15	4	−0.033	−4.79
101	LAWYQQKPGKAPKL	15	12	52	45.75	8.84	103.95	8	0.157	5.45
102	MHWVRQAPGQGLEWM	14	15	56	54.50	10.54	130.27	4	−0.106	0.04
103	VSWYQQHPGKAPKL	15	13	52	50.45	9.59	113.81	8	0.110	4.76
104	GDRVTITCRASQGIS	14	15	58	51.80	8.68	134.40	0	0.007	−2.06
105	GASVKVSCKASGYTF	15	13	53	44.05	8.86	116.11	0	0.115	−2.48
106	EEQYNSTYRVVSVLTVLHQDW	21	24	89	82.80	12.81	182.90	0	−0.081	−5.22
107	LMIYE	5	6	23	18.25	4.47	38.13	0	−0.135	0.19
108	NTLYL	5	5	21	17.95	3.86	39.21	0	−0.023	0.40
109	NSTYR	5	5	24	20.65	1.38	38.43	0	0.062	3.08
110	PAVLQ	4	4	13	15.00	4.85	38.40	4	−0.005	2.02
111	VDTSK	6	6	22	18.40	2.43	48.87	0	0.029	2.06
112	KVYAC	5	4	19	13.25	3.09	28.56	0	0.024	0.87
113	MHWVR	5	5	23	19.50	3.89	45.42	0	−0.124	0.43
114	LNNFY	5	5	21	18.75	3.34	39.18	0	0.051	0.92
115	VHWYQ	5	5	21	20.25	3.16	37.21	0	−0.058	1.12
116	GTDFT	5	6	18	17.40	3.04	48.96	0	−0.054	1.12
117	PGLVR	5	5	17	17.50	5.49	47.26	4	−0.048	2.26
118	GGLVQ	5	5	16	17.50	4.23	48.42	0	0.043	−0.63
119	WSWIR	5	5	23	17.70	3.43	45.05	0	−0.140	0.89
120	WSWVR	5	5	22	17.70	3.44	45.08	0	−0.123	0.81
121	GGLVQ	5	5	16	17.50	4.23	48.42	0	0.043	−0.63
122	LAWYQ	4	4	18	15.25	2.69	27.72	0	−0.064	1.19
123	MHWVR	5	5	23	19.50	3.89	45.42	0	−0.124	0.43
124	GDRVT	5	6	20	18.70	2.69	48.02	0	−0.035	1.66
125	GASVK	5	4	16	13.70	2.53	38.72	0	0.081	0.88
126	EEQYN	5	7	24	23.75	0.57	39.01	0	0.107	4.32
127	VSNRP	5	5	19	19.70	3.83	47.65	4	0.061	4.13
128	QMNSL	5	5	22	20.20	3.04	48.18	0	0.084	1.16
129	VVSVL	5	5	17	15.70	6.18	48.63	0	−0.075	0.00
130	SSGLY	5	5	19	17.15	3.22	39.12	0	−0.018	0.89
131	NQFSL	5	5	21	19.70	3.28	48.73	0	0.079	0.84
132	EVTHQ	5	6	21	22.20	2.49	48.40	0	0.031	1.92
133	QAPGK	5	4	15	16.00	2.66	38.18	4	0.134	4.63
134	PREAK	5	5	20	17.50	2.05	37.37	4	0.054	6.16
135	QKPGQ	6	5	20	21.50	2.70	47.81	4	0.195	5.36
136	LTISS	5	5	20	17.10	4.11	48.93	0	−0.041	−0.08
137	PSQTL	5	5	18	19.40	4.25	48.41	4	0.029	3.59
138	PGGSL	5	5	13	16.20	4.98	48.32	4	0.004	2.10
139	QPPGK	6	5	16	19.50	4.55	47.54	8	0.122	7.10
140	QLPGT	5	5	16	18.70	4.53	48.25	4	0.032	3.08
141	QPPGR	5	5	16	20.50	4.53	46.86	8	0.054	7.11
142	PGRSL	5	5	18	18.20	4.39	47.43	4	−0.015	3.47
143	QKPGK	7	5	22	20.00	2.73	47.66	4	0.201	5.51
144	QAPGQ	4	4	13	17.50	2.63	38.32	4	0.128	4.24
145	QHPGK	6	5	19	21.00	3.11	47.70	4	0.122	4.86
146	ITCRA	4	4	19	14.20	3.14	37.69	0	−0.031	0.60
147	VSCKA	5	4	18	13.20	3.04	38.41	0	0.069	0.62
148	STYRV	5	5	23	19.15	2.79	38.18	0	−0.034	1.62

*Sequence* sequence named according to IUPAC recommendations (IUPAC 1983), *AM* number of non-conjugated amine groups, *AC* number of carboxylic acid groups, *ROT* number of non-trivial (not CX3), non-hindered (not alkene, amide, small ring) rotatable bonds, *HBA* hydrogen bond acceptors, *QPCaco* predicted apparent Caco-2 cell permeability, *IP* ionization potential, *eV* electronovolts, *NON* number of ring atoms not able to form conjugated aromatic systems, *QPlogS* solubility, *AEx* arithmetic expression value *Ln*(*AM* − *IP* + *AC* × *ROT*) − (*QPCaco* − *NON*)

The shortest sequences in the APR group consisted of 5 amino acids. 46.4 % of APR sequences consisted of only 5 amino acids. A CS was created also based on the peptides with the length of 5 amino acids. From each tregitope (*n* = 22) two sequences were chosen (Table 1; sequences 85–106). The first one was made of the first five amino acids of each tregitope (1–5) and second one was made of the next consecutive five amino acids of each tregitope (6–10). This way, CS sequences (*n* = 44) of the length of 5 amino acids each were obtained. One of the sequences from the CS was removed from the analysis (VVSVL). This sequence was the same as one of the APR sequences. Another one (VSWYQ) was also removed from the CS group as a result of double selection from the group of tregitopes during conducted procedures. This way, the final number of CS sequences was 42 (Table 1, sequences 107–148).

Tregitopes were used to build CS as they are short amino acid sequences present in the structure of many therapeutic proteins. After protein internalization, these sequences are responsible for the modulation of an immune response by influencing the regulatory T cells. The effect of tregitope presentation by MHC-II is a tolerogenic action (De Groot et al. 2013). The presence of tregitopes in therapeutic protein sequences (except vaccines) is a desired element considering the suppression of immune response in relation to the administered protein.

### Physicochemical parameters calculations

In the first phase of physicochemical characterization of analyzed sequences physicochemical parameters of single amino acids were calculated. 16 parameters were taken from PubMed® database (XLogP3, rotatable bond count, heavy atom count, formal charge, complexity, isotope atom count, defined atom stereocenter count, undefined atom stereocenter count, defined bond stereocenter count, undefined bond stereocenter count, covalently-bonded unit count). Analysis of 51 physicochemical parameters of single amino acids was completed using QikProp 3.1 from Schrödinger package (v 31207) software (Grabowski et al. 2012). QikProp was run in the normal mode. Three-dimensional structures of compounds were prepared in LigPrep 2.2 using settings recommended in the QikProp’s user manual (Schrödinger 2015). In the initial phase of study, 62 parameters and features of physicochemical structure were used. They were calculated separately for each amino acid that was a part of the examined sequences.

In the second phase, physicochemical parameters for whole sequences were calculated. In this phase arithmetic expression value (AEx) was created with the use of eight clue physicochemical parameters (Table 1). In cases of such parameters as: number of non-conjugated amine groups (AM), number of carboxylic acid groups (AC), number of non-trivial (not CX3), non-hindered (not alkene, amide, small ring) rotatable bonds (ROT), number of ring atoms not able to form conjugated aromatic systems (NON), ionization potential (IP) and hydrogen bond acceptors (HBA), the sum of the values of particular parameter (SP) calculated for each amino acid separately *SP* = (*SP*_*i*_ = *n*_*i*_) + (*SP*_*ii*_ × *n*_*ii*_) + (*SP*_*iii*_ × *n*_*iii*_) …, where SP_i-iii_ – structure parameter calculated for particular amino acid, n – amount of particular amino acid in the sequence, i, ii, iii – particular amino acids was calculated.

In case of predicted apparent Caco-2 cell permeability (QPCaco) and solubility (QPlogS), SP was an arithmetic mean of particular physicochemical parameters calculated according to the formula:

$$ SP=\left[\left(S{P}_i\times {n}_i\right)+\left(S{P}_{ii}\times {n}_{ii}\right)+\left(S{P}_{iii}\times {n}_{iii}\right)\dots \right]/N, $$

where N – number of non-replayed amino acids in sequence. This way calculated physicochemical parameters (Table 1) were used to search for the correlation that could differentiate APR from CS.

### Creation of arithmetic expressions

The search for differences in each SP between the APR and CS groups (Table 1) did not yield any significant findings. Therefore, an attempt to create an arithmetic expression value (AEx) consisting of several different SP was made. To this end, the method published earlier was used (Grabowski et al. 2012). Many compilations of SP were tested (not published data), as the result of which the arithmetic statement was distincted: *Ln*(*AM* − *IP* + *AC* × *ROT*) − (*QPCaco* − *NON*). A value QPlogS and this arithmetic statement were used to classify the sequence groups and to exhibit a significance of differences between classes (APR and CS). QPlogS is a physicochemical parameter of a complex character. This focuses the information of solubility in water. However, this information combines many properties linked to a molecule solubility and its electrostatical character. Hence, there was an attempt to use that parameter in presented model.

### Statistical analysis and model validation

Statistical analysis was performed with the use of GraphPad Prism 6.0 software. All relationships were confirmed by Mann-Whitney test (Z_c_ statistics) and differences with *p* <0.05 were regarded as statistically significant. Arithmetic mean (M), standard deviation (SD), lower and higher 95 % confidence intervals for M (CI low, CI high), and standard error (SE) was calculated ($SD/\sqrt{N}$, where – N is total number of sequences (APR and CS)). Sample size of training set (APR) was positively verified by Toplis ratio (ratio of the number of chemicals in the training set to the number of descriptors in the AEx is >5:1) (OECD 2007; ECHA 2008).

Classification model and four classes (A, B, C, D) of sequences were finally presented. Currently, the Cooper statistics is the most widely used method of classification model validation (Fang and Fang 2013; Fang et al. 2013; Zambrano et al. 2015). That is why, for model validation Cooper statistics based on Bayesian approach (sensitivity – S_n_, specificity – S_p_, accuracy – A_c_, error rate – E_r_, positive predictivity – P_p_, negative predictivity – N_p_, false positive (over-classification) rate – FP_oc_, false negative (under-classification) rate – FN_uc_, proportion of active chemicals in a population – P_as_) was presented. Cooper statistics was calculated using equations: *S*_*p*_ = *T*_*n*_/(*T*_*n*_ + *F*_*p*_), *S*_*n*_ = *T*_*p*_/(*T*_*p*_ + *F*_*n*_), *A*_*c*_ = (*T*_*p*_ + *T*_*n*_)/*N* where *E*_*r*_ = [*N* − (*T*_*p*_ + *T*_*n*_)]/*N*, *P*_*p*_ = *T*_*p*_/(*T*_*p*_ + *F*_*p*_), *N*_*p*_ = *T*_*n*_/(*T*_*n*_ + *F*_*n*_), *FP*_*oc*_ = *F*_*p*_/(*F*_*p*_ + *T*_*n*_), *FN*_*uc*_ = *F*_*n*_/(*F*_*n*_ + *T*_*p*_), *P*_*as*_ = *T*_*n*_/(*T*_*n*_ + *F*_*p*_), where T_p_ (true positive) is the number of compounds correctly classified as APR, T_n_ (true negative) is the number of compounds correctly classified as CS, F_n_ (false negative) is the number of APR compounds classified as CS, F_p_ (false positive) is the number of CS compounds classified as APR and N is total number of sequences (APR and CS). Each calculated value was multiplied by 100 and expressed as %, model was verified as validated if Cooper statistics is significantly greater than 50 % (OECD 2007).

## Results

The mean values of SP calculated for particular groups of sequences were presented in Table 2 (Table 2).

**Table 2:** An arithmetic mean and standard deviations of physicochemical parameters used for arithmetic expression (AEx; *Ln*(*AM* − *IP* + *AC* × *ROT*) − (*QPCaco* − *NON*)) determination

Analyzed sequences	Arithmetic mean; standard deviation								
	AM	AC	ROT	HBA	QPCaco [nm/s]	IP [eV]	NON	QPlogS	AEx
Aggregation prone regions	6.0; 2.0	6.0; 2.0	23.0; 7.0	19.64; 6.01	5.84; 2.17	49.70; 13.96	0.0; 1.0	−0.095; 0.095	−1.21; 2.02
Tregitopes	17.0; 3.0	17.0; 4.0	63.0; 14.0	58.98; 12.61	11.83; 2.88	145.77; 32.73	4.0; 2.0	−0.003; 0.113	−0.68; 3.40
Control sequences	5.0; 1.0	5.0; 1.0	19.0; 3.0	18.17; 2.30	3.44; 1.04	43.27; 5.65	2.0; 2.0	0.015; 0.084	2.23; 2.03

*AM* number of non-conjugated amine groups, *AC* number of carboxylic acid groups, *ROT* number of non-trivial (not CX3), non-hindered (not alkene, amide, small ring) rotatable bonds, *HBA* hydrogen bond acceptors, *QPCaco* predicted apparent Caco-2 cell permeability, *IP* ionization potential, *eV* electronovolt, *NON* number of ring atoms not able to form conjugated aromatic systems, *QPlogS* solubility

**Fig. 1:**
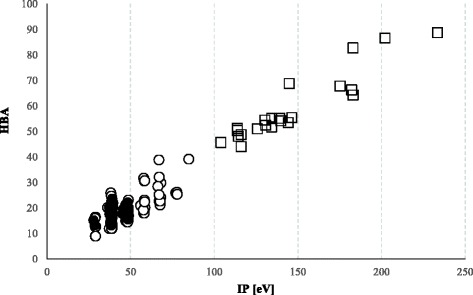
A relationship between hydrogen bond acceptors (HBA) and ionization potential (IP-[eV – electronovolts]). *APR* aggregation prone regions (○; *n* = 84); tregitope sequences (□; *n* = 22), *CS* control set extracted from tregitopes (●; *n* = 42)

**Fig. 2:**
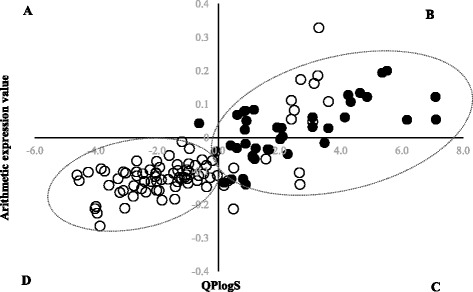
A separation of 4 classes (**a**, **b**, **c**, **d**) of dependencies and the relationship between aqueous solubility (QPlogS) and arithmetic expression value (AEx), *Ln*(*AM* − *IP* + *AC* × *ROT*) − (*QPCaco* − *NON*). *APR* aggregation prone regions (○; *n* = 84), *CS* control set extracted from tregitopes (●; *n* = 42), *AC* number of carboxylic acid groups, *AM* number of non-conjugated amine groups, *IP* ionization potential, *ROT* number of non-trivial (not CX3), non-hindered (not alkene, amide, small ring) rotatable bonds, *QPCaco* predicted apparent Caco-2 cell permeability, *NON* number of ring atoms not able to form conjugated aromatic systems

At the initial stage of study, the differences in SP between APR and CS were searched. As the result, they could differentiate significantly between these two groups. The analysis of single parameters did not yield its expected results. In case of comparative analysis of SP calculated for the tregitope sequences and APR, significant differences were identified (*p* <0.05), for instance in relation to HBA↔IP. After a selection of shorter sequences (CS) from the same tregitope sequences, though, it turned out that the differences in relation to HBA↔IP were not significant (Fig. 1). The significant differences (*p* <0.05) in relation to values SP of tregitopes and APR were also stated for correlations: FISA↔*AC* × *DN*^0.5/*SA*^, FISA↔Vol, Vol↔HBA, QPlogS↔FISA, where FISA – hydrophilic component of the solvent accessible surface area, *AC* × *DN*^0.5/*SA*^ – index of cohesive interaction in solids, Vol – total solvent accessible volume in cubic angstroms (Å^2^) using a probe with a 1.4 Å radius. However, all the same correlations were not significant for the CS selected from the tregitopes. At the next stage of study, SP was used to create arithmetic statement (AEx) that allowed differentiation of APR (*n* = 84) from CS (*n* = 42) with a sensitivity of 79.76 %. After statement of a correlation AEx↔QPlogS, the sequences APR and CS were differentiated on 4 different classes (A, B, C, D). The range of classes are characterized with the values of parameters QPlogS and AEx. A definition of class includes the values: QPlogs > 0 and AEx < 0 (class A), QPlogs ≥ 0 and AEx > 0 (class B), QPlogs < 0 and AEx ≥ 0 (class C), QPlogs ≤ 0 and AEx ≤ 0 (class D), (Fig. 2). A range specific for APR illustrates class D on Figure 2. As a result of using AEx, only 20.24 % of APR were incorrectly recognized as sequences not connected with the aggregation process (class B and C on Fig. 2). And only one of 42 CS sequences was recognized as a sequence potentially dangerous and classified to class D. As a result, 97.67 % of CS sequences were classified as not possessing any features connected with forming the aggregates (Table 3) – class A, B, C on the Figure 2.

**Table 3:** Summary of statistics for arithmetic expression value (AEx) *Ln*(*AM* − *IP* + *AC* × *ROT*) − (*QPCaco* − *NON*) and solubility (APlogS) of each sequence (aggregation prone regions and control set) in classes A, B, C, D

	Class							
	A		B		C		D	
	Aggregation prone regions							
Parameters	AEx	QPlogS	AEx	QPlogS	AEx	QPlogS	AEx	QPlogS
M	na.	na.	0.313	0.015	0.103	−0.012	−1.629	−0.098
SD	na.	na.	0.420	0.082	0.990	0.041	1.167	0.044
CI low	na.	na.	0.039	−0.039	−0.583	−0.041	−1.909	−0.108
CI high	na.	na.	0.587	0.068	0.789	0.017	−1.350	−0.087
n	0		9		8		67	
SE	na.	na.	0.140	0.027	0.350	0.015	0.143	0.005
	Control set sequences							
M	−0.029	0.002	1.733	0.041	0.472	−0.025	−0.002	−0.001
SD	0.000	0.000	2.060	0.052	0.801	0.046	0.000	0.000
CI low	na.	na.	0.873	0.019	0.091	−0.046	na.	na.
CI high	na.	na.	2.594	0.062	0.853	−0.003	na.	na.
n	2		22		17		1	
SE	na.	na.	0.439	0.011	0.194	0.011	na.	na.

*Groups* groups of parameters depicted in Fig. 2, *M* arithmetic mean, *SD* standard deviation, *CI low* lower 95 % confidence interval for M, *CI high* higher 95 % confidence interval for M, *n* number of sequences observed in specific class (A, B, C, D), *SE* standard error, *na.* not applicable, QPlogs >0 and AEx <0 (class A), QPlogs ≥0 and AEx >0 (class B), QPlogs <0 and AEx ≥0 (class C), QPlogs ≤0 and AEx ≤0 (class D)

Out of 127 sequences (a sum of APR and CS) only one was present in A class. 67 APR sequences were classified to class D. Other sequences were in classes: B and C. The proposed classification model did not allow total separation of APR from CS. During analyses, it turned out that four out of 42 CS sequences had regions that were repeated in APR. These regions contained hydrophobic amino acids such as F, I, L, M and N. These regions were: LMI, LYL, TDF and QYN. Only one APR (CQQYN) was classified to class B instead of class D. In relation to CS, none of mentioned regions (LMI, LYL, TDF, QYN) impacted on incorrect classification of CS. Every CS sequence possessing the mentioned regions in its structure was assigned to class C (EEQYN) or B (LMIYE; NTLYL; GTDFT).

Significant difference (*p* <0.05) between AEx value calculated for APR (*n* = 84) and CS (*n* = 42), Z_c_ = 7.172 was stated. Moreover, significant difference (*p* <0.05) was also stated between QPlogS value calculated for APR (*n* = 84) and QPlogS calculated for CS (*n* = 42) *p* <0.05, Z_c_ = 6.270. Calculated Cooper statistics was S_n_ = 79.76 %, S_p_ = 97.62 %, A_c_ = 85.71 %, E_r_ = 14.29 %, P_p_ = 98.53 %, N_p_ =70.69 %, FP_oc_ = 2.38 %, FN_uc_ = 20.24 % and P_as_ = 85.71 %.

## Discussion

This study attempted to use the physicochemical parameters of single amino acids to detect APR sequences in therapeutic proteins. The introduced method involved analysis using software used previously mostly for calculations of physicochemical parameters of small molecules. This method uses the analysis of physicochemical parameters of single amino acids and bases on a prediction of final parameter (SP). This parameter, in turn, is the basis for creating a sequence or region characterization based on AEx, constructed of many SP (*AEx = SP* ⟺ *SP* ⟺ *SP….*, where ⟺ means mathematic operation). AEx with QPlogS was used to construct a model, where 4 sequence classes were defined. Class D includes APR sequences, and classes A, B, C – sequences that do not have the same influence on aggregation bridges forming.

In the course of the study, it was stated that using the long amino acid sequences to verify the presented model implemented false positives. In long tregitope sequences AEx had a value significantly different from AEx calculated for APR. However, this may result from the existence of feature camouflage of the shorter CS (CS derived from tregitopes, *n* = 5).

The study proposes a classification model of APR consisting in a separation of APR based on the differences in the value of QPlogS and AEx in relation to sequences that do not form aggregates. A value of water solubility or hydrophobicity of APR with reference to APR has been discussed in many studies (Wang et al. 2009; Tsolis et al. 2013; Zbilut et al. 2003; Wu et al. 2014). The significance of this feature in relation to APR was also confirmed in this study. Moreover, it was stated that the charge characterization of particular amino acids present in analyzed sequences has a significant correlation with APR. It is indicated by the presence in AEx of such parameters as: ionisation potential, number of amine groups or number of carboxylic acid groups. At least three parameters used to construct AEx relate to the charge characterization of analyzed sequences. The presence of IP in AEx does not seem to be accidental. The IP value is determined, among other things, in relation to the oxidative potential of amino acids. It is known that IP value is connected with the proton-donating or proton-accepting character of the amino group and carbonyl groups of amino acids (Hirakawa 2014). IP is a parameter indicative of the molecular ability to transfer positive ion. Therefore IP is connected with the oxidative reactions of amino acids (Rooman and Wintjens 2014). On the other hand, oxidation of some amino acids (histidine, methionine, cysteine, tryptophan, tyrosine) may have influence on the increase of aggregation forming dynamics (Li et al. 1995).

Although some sequences were not classified correctly, validation parameters confirm the predictive quality of the model. Based on the calculations, it can be deduced that the finding of APR in the protein structure with the use of parameters used so far for small molecules is possible. The study confirmed previous observations concerning the influence of short, hydrophobic protein sequences on the initiation of the protein aggregation process. Additionally, the significant share of electrostatistic parameters including IP in relation to classification parameters was indicated.

## Conclusions

The study proposes a classification model of APR consisting in a distinction of APR based on the differences in the structure in relation to sequences that do not form aggregates. Key parameters for validation of the presented model include: number of non-conjugated amine groups, number of carboxylic acid groups, number of non-trivial (not CX3), non-hindered (not alkene, amide, small ring) rotatable bonds, hydrogen bond acceptors, predicted apparent Caco-2 cell permeability, ionization potential, number of ring atoms not able to form conjugated aromatic systems and solubility.

This presented model allows selection of APR’s in the protein sequence in non-clinical drug development process.

## Ethics approval and consent to participate

Not applicable.

## Consent for publication

Not applicable.
